# Phylogenetic Analysis of Enterohemorrhagic *Escherichia coli* O157, Germany, 1987–2008

**DOI:** 10.3201/eid1604.091361

**Published:** 2010-04

**Authors:** Christian Jenke, Dag Harmsen, Thomas Weniger, Jörg Rothgänger, Eija Hyytiä-Trees, Martina Bielaszewska, Helge Karch, Alexander Mellmann

**Affiliations:** Institute for Hygiene, Münster, Germany (C. Jenke, M. Bielaszewska, H. Karch, A. Mellmann); Department of Periodontology, Münster (D. Harmsen, T. Weniger); Ridom GmbH, Würzburg, Germany (J. Rothgänger); Centers for Disease Control and Prevention, Atlanta, Georgia, USA (E. Hyytiä-Trees)

**Keywords:** Enterohemorrhagic Escherichia coli, EHEC O157, MLVA, phylogeny, sorbitol fermentation, bacteria, typing, Germany, research

## Abstract

Multilocus variable number tandem repeat analysis (MLVA) is a subtyping technique for characterizing human pathogenic bacteria such as enterohemorrhagic *Escherichia coli* (EHEC) O157. We determined the phylogeny of 202 epidemiologically unrelated EHEC O157:H7/H^–^ clinical isolates through 8 MLVA loci obtained in Germany during 1987–2008. Biodiversity in the loci ranged from 0.66 to 0.90. Four of 8 loci showed null alleles and a frequency <44.1%. These loci were distributed among 48.5% of all strains. Overall, 141 MLVA profiles were identified. Phylogenetic analysis assigned 67.3% of the strains to 19 MLVA clusters. Specific MLVA profiles with an evolutionary persistence were identified, particularly within sorbitol-fermenting EHEC O157:H^–^.These pathogens belonged to the same MLVA cluster. Our findings indicate successful persistence of this clone.

## Abstract 2

Clones of this organism persisted during the study period.

Enterohemorrhagic *Escherichia coli* (EHEC) O157:H7 infections have substantial medical, public health, and economic effects (*1*,*2*). Most symptomatically infected patients have painful bloody diarrhea (*2*,*3*). Hemolytic uremic syndrome (HUS) develops in ≈15% of infected children ≈1 week after the first loose stool. HUS is a thrombotic microangiopathy and consists of nonimmune hemolytic anemia, thrombocytopenia, and renal failure (*1*). Currently, HUS is the main cause of acute renal failure in children (*4*). In Germany, *E*. *coli* O157:H7, which is the most frequent EHEC serotype implicated in HUS, is not the only relevant EHEC O157 involved. Sorbitol-fermenting (SF) *E*. *coli* O157:H^–^ (nonmotile) strains cause ≈20% of all cases of HUS (*5*). Unlike *E*. *coli* O157:H7, organisms within this clone can ferment sorbitol after overnight incubation on sorbitol MacConkey agar. Although EHEC O157:H7 causes a zoonotic disease mainly associated with cattle, efforts to determine the animal reservoir of SF EHEC O157:H^–^ have been unsuccessful (*5*).

To identify reservoirs of EHEC O157:H7 infections and of other foodborne pathogens and to elucidate the molecular epidemiology of these pathogens in the United States, PulseNet was established in 1996 (*6*). This US national molecular subtyping network for foodborne disease surveillance facilitates subtyping of bacterial foodborne pathogens for epidemiologic purposes. This network is based on characterization of whole bacterial genomes by using macrorestriction digestion patterns that are separated by pulsed-field gel electrophoresis (PFGE), a technique that has emerged as a common standard for subtyping EHEC O157 isolates (*6*). Despite its high discriminatory power, PFGE can be problematic because it requires great efforts to ensure intralaboratory and interlaboratory reproducibility (*7*–*10*). Furthermore, its application is labor-intensive and difficult to automate. Thus, this technique can be biased by subjective interpretation of band patterns (*7*,*8*). In addition, band patterns can be altered by the presence of mobile genetic elements.

To overcome these drawbacks, other molecular methods were developed, among them multilocus variable number tandem repeat (VNTR) analysis (MLVA). MLVA is based on the characterization of different VNTR regions throughout the bacterial genome. Repeat regions are amplified by using PCRs, and resulting fragments are sized to determine the number of repeats. The combination of numbers of repeats of different VNTR loci results in an allelic profile known as the typing result. First developed in 1995 for *Mycobacterium tuberculosis* (*11*), MLVA is now a common typing method for an increasing number of pathogens (*12*,*13*). For EHEC O157, different MLVA schemes with some overlaps of VNTR regions have been published and have demonstrated a capability to detect outbreaks and differentiate closely related EHEC O157 isolates not discriminated by PFGE (*8*,*14*,*15*). These findings qualify MLVA as the second-generation subtyping method for PulseNet (*8*).

In addition to its use in infectious disease surveillance, MLVA also can be used to study phylogeny of pathogens, especially recently evolved clonal pathogens such as *M*. *tuberculosis* (*16*,*17*) or *Bacillus anthracis* (*18*). However, because of limited diversity in their housekeeping genes, which are the genomic targets for phylogenetic investigations based on multilocus sequence typing (MLST), the common technique for phylogenetic studies (*19*,*20*), certain monomorphic organisms could not be sufficiently differentiated by MLST (*16*,*18*). Similarly, EHEC O157 lacks diversity in its housekeeping genes (*21*,*22*), which hampers phylogenetic analysis of EHEC O157 by MLST.

We investigated the phylogeny of EHEC O157:H7 and SF EHEC O157:H^–^ strains isolated during 1987–2008 in Germany by applying the current PulseNet MLVA protocol for *E*. *coli* O157 (*23*). The purpose of our study was to gain a deeper insight into the evolution and spread of this pathogen since 1987, when the first cases of EHEC O157 infections were detected (*24*,*25*).

## Materials and Methods

### Clinical Isolates

Up to 17 epidemiologically unrelated EHEC O157:H7/H^–^ isolates per year obtained during 1987–2008 were randomly selected from the strain collection of the Institute of Hygiene and the National Consulting Laboratory on HUS, University Hospital Münster, Germany. All 202 O157 strains (61 of which were SF EHEC O157:H^–^) were isolated from humans, including patients with HUS (145), bloody diarrhea (12), or diarrhea without visible blood (40), and asymptomatic carriers (5) during epidemiologic investigations. Isolates were obtained from areas throughout Germany. Procedures used for detecting and isolating EHEC O157 from stool samples were described (*26*,*27*). Isolates were confirmed as *E*. *coli* by the API 20 E test (bioMérieux, Marcy l’Etoile, France) and serotyped by using antisera against *E*. *coli* O antigens 1–181 and H antigens 1–56 (*28*). Subtyping of *fliC* genes in nonmotile isolates by using *Hha*I restriction fragment length polymorphism of amplicons obtained with primers FSa1 and rFSa1 (*29*,*30*) confirmed the presence of *fliC*H7 in all isolates. EHEC O157:H7 strain EDL933 (*31*,*32*) was used as a reference strain in all analyses.

### MLVA of EHEC O157

Strains were grown overnight on Columbia blood agar (Heipha; Eppelheim, Germany) at 37°C. A loop of a fresh culture was suspended in 100 μL of Chelex-100 solution (Bio-Rad, Hercules, CA, USA) and vortexed briefly. After boiling and thorough mixing, samples were centrifuged and DNA-containing supernatants were stored at –20°C until use. To calibrate sequencer-specific variation of fragment length, the exact number of repeats of reference strain O157:H7 EDL933 was initially determined in silico on the basis of its genome sequence (reference sequences NC_002655 [chromosome] and NC_007414 [plasmid]; National Center for Biotechnology Information [NCBI], Bethesda, MD, USA) by using Tandem Repeats Finder software (*33*). Subsequently, the length of the in silico–determined repeats was subtracted from the fragment length of each respective VNTR locus generated in 8 independent capillary electrophoresis runs of strain EDL933 to determine the offset (primer plus VNTR-flanking regions). This locus-specific offset was then used to calculate the correct number of repeats of unknown isolates. Fragments for MLVA typing were generated in 2 multiplex PCRs comprising either VNTR loci 3, 9, 25, and 34 (multiplex 1) or VNTR loci 17, 19, 36, and 37 (multiplex 2) (Table A1), according to the current PulseNet MLVA protocol for *E*. *coli* O157 (*23*).

PCR amplification was performed in a reaction mixture of 10 μL containing 5 μL of Type-it Multiplex Master Mix (QIAGEN, Hilden, Germany), ≈30 ng of DNA template, and VNTR-specific primers for each of the 4 VNTR loci. Concentration, primer sequences, and respective dyes used are shown in Table A1. PCRs were performed and prepared for subsequent analysis on sequencers in accordance with the manufacturer’s instructions (Table A1). PCR products were diluted 1:10 with water purified by high performance liquid chromatography, and 1.0 μL of diluted DNA was mixed with 13.7 μL of HiDi formamide (Applied Biosystems, Foster City, CA, USA) and 0.3 μL of GeneScan-600 LIZ Size Standard (Applied Biosystems) as internal lane size standard. Before fragment sizing in the ABI Prism 3130xl Genetic Analyzer System (Applied Biosystems), samples were incubated for 5 min at 95°C and immediately frozen at –20°C for >3 min to denature the DNA.

If a VNTR locus was not detected during fragment analysis, reactions were repeated by using singleplex reactions with minor modifications to amplify the specific locus. In that particular instance, the primer concentration was increased to 0.2 µmol/L, annealing temperatures were reduced to 55°C, and the extension time was tripled to amplify larger fragments because of possible insertion sequence element transposition or other genetic events. Subsequently, fragments were characterized by using standard agarose gel electrophoresis. If the fragment was larger than the usual range of fragment sizes of the corresponding VNTR, the PCR product was sequenced.

### Data Analysis

After fragment analysis, corresponding peak data were examined by using GeneMapper 4.0 software (Applied Biosystems) to calculate the repeat number for each VNTR locus on the basis of fragment length. Partial repeats were rounded to the closest repeat number in accordance with the current Centers for Disease Control and Prevention (CDC) (Atlanta, GA, USA) MLVA O157 protocol (*23*). If >1 amplicon for a specific VNTR locus was detected and the size difference matched >1 repeat lengths (so-called stutter peaks), the one with the highest fluorescence level was used to calculate the repeat number. A null allele was assigned if either no amplicon was detected or agarose gel electrophoresis data showed an amplicon of a size that was beyond the usual range of fragment size of the specific VNTR locus. Corresponding alleles were designated as –2. In the hypothetical situation in which an amplicon without the repeat region was detected, it was designated as –1 (*8*). Null alleles were also included in the overall number of alleles in a specific VNTR locus.

Index of diversity (ID) (*34*) and typeability were calculated by using EpiCompare 1.0 software (Ridom GmbH, Würzburg, Germany). A minimum spanning tree (MST) was generated by using SeqSphere software 0.9 β (Ridom GmbH). All MLVA profiles that differed at <2 alleles were grouped as an MLVA cluster. To determine the cluster-defining profile of clusters containing >2 MLVA profiles, the MST priority rule (that the profile with the highest number of single locus variants is chosen) was applied.

Significance of associations of MLVA profiles or clusters comprising >4 strains with clinical outcome (HUS vs. non-HUS) were calculated by using a χ^2^ test with Yates correction (EpiInfo 6 software; CDC) when appropriate. p values <0.05 were considered significant.

## Results

### EHEC O157 Strains and VNTR Loci Characterization

The 202 EHEC O157 strains showed 141 MLVA profiles. Of these profiles, MLVA profile 4/8/-2/2/3/9/-2/5 was the most common and was present in 30 isolates. In contrast, 122 profiles were detected only once. Detailed characteristics of the different VNTR loci identified in this study are shown in Table 1. The number of alleles for the VNTRs ranged from 6 (VNTR-25) to 22 (VNTR-3). Calculation of the ID resulted in values from 0.66 (VNTR-34) to 0.9 (VNTR-9). Half of the VNTR loci (VNTR-3, VNTR-9, VNTR-36, and VNTR-37) showed null alleles with different frequencies ranging from 2.0% (VNTR-3 and VNTR-37) to 44.1% (VNTR-36).

**Table 1:** VNTR characteristics of enterohemorrhagic *Escherichia coli*, Germany, 1987–2008*

VNTR locus	Alternative name†	Repeat length, bp	Inside ORF (no.)‡	No. repeats		No. alleles§	Null alleles	Null allele frequency, %	ID	Typeability, %¶
				Minimum	Maximum					
3	Vhec3, TR5	6	+ (Z0268)	3	23	22	+	2.0	0.86	98.0
34	Vhec2, TR6	18	+ (Z5865)	4	12	8	–	–	0.66	100.0
9	Vhec4, TR1	6	+ (Z3935/Z3936)	6	23	15	+	31.7	0.90	68.3
25	TR4	6	–	2	15	6	–	–	0.74	100.0
17	TR3	6	+ (Z5935)	2	19	10	–	–	0.80	100.0
19	TR7	6	+ (Z3274)	3	11	9	–	–	0.76	100.0
36#	Vhec7	7	–	3	15	13	+	44.1	0.87	55.9
37#	–	6	+ (L7083)	3	17	11	+	2.0	0.82	98.0

*VNTR, variable number tandem repeat; ORF, open reading frame; ID, index of diversity without null alleles. †Vhec loci as explained by Lindstedt et al. (*15*). TR loci are from Noller et al. (*14*). ‡Number is based on current EDL933 genome data. ORF encoding VNTR loci encoded either hypothetical proteins or proteins with unknown function. §Including null alleles. ¶Typeability determines the proportion of all alleles without null alleles. #Located on plasmid pO157 of reference strain EDL933.

To determine whether amplification failure caused by mutations in primer-binding regions or by complete deletions of the VNTR region were the reason for these null alleles or the insertion of fragments such as mobile genetic elements resulted in larger (and therefore by capillary electrophoresis) undetectable fragments, the respective fragments were analyzed by using standard gel electrophoresis. In some cases, large fragments (>1.3 kb) were detected. Sequence analysis and an NCBI nucleotide BLAST search (http://blast.ncbi.nlm.nih.gov/Blast.cgi) of randomly selected samples indicated the presence of insertion sequence elements of the IS3 family. The typeability of different VNTR loci ranged from 55.9% to 100% (Table 1). Null alleles were present in 48.5% of the strains (98/202). The overall ID of all MLVA profiles was 0.98.

### MLVA Clustering and Phylogeny

Comparison of MLVA profiles assembled 136 of the 202 strains (67.3%) into clusters sharing >6 of the 8 VNTR loci. This grouping resulted in 19 MLVA clusters consisting of 2–61 isolates comprising 81 MLVA profiles. The remaining 66 strains (32.7%), which had 60 MLVA profiles, could not be associated with any cluster (Table 2). Strains within clusters consisting of >4 MLVA profiles were isolated over a period >7 years. The 61 strains of cluster 1 were isolated during 1988–2008. Among these strains, the cluster-defining MLVA profile 4/8/-2/2/3/9/-2/5 was the most common profile. It included 30 (14.9%) of 202 strains isolated during 1988–2008. The profile of the same cluster (cluster 1) with the second highest number of strains differed only in VNTR-37 with an additional repeat and included 5.0% of the strains (10/202). The corresponding strains were isolated during 1995–2008 (Table 2; Figure).

**Table 2:** MLVA cluster profile of enterohemorrhagic *Escherichia coli* O157, Germany, 1987–2008*

MLVA cluster	Cluster-defining MLVA profile	No. strains (% sorbitol-fermenting)	No. MLVA profiles	Years of strain isolation
1	4/8/-2/2/3/9/-2/5	61 (100)	16	1988, 1989, 1993, 1995–2008
2	7/7/13/4/5/7/-2/7	11 (0)	7	1996–1998, 2000, 2002, 2005, 2006
3	11/8/10/3/5/6/8/3	9 (0)	8	2001, 2004, 2005, 2007, 2008
4	18/7/8/3/5/7/-2/7	8 (0)	8	1996–1999, 2001, 2005, 2006
5	5/7/13/5/6/6/6/6	7 (0)	5	1991, 1999, 2007
6	12/7/16/3/5/6/5/7	5 (0)	5	1990, 1992, 1999, 2001, 2004
7	18/7/10/4/4/9/4/9	5 (0)	4	1996, 1998, 2001–2003
8	9/7/9/4/6/7/9/8	3 (0)	3	2007, 2008
9	13/7/11/4/4/8/4/8	3 (0)	3	1992, 1993, 2002
10	6/6/16/3/6/6/6/6	3 (0)	3	2005, 2006
11	11/9/10/5/7/6/11/7	3 (0)	3	1996, 2000, 2007
12	8/10/13/6/7/7/5/6	3 (0)	2	2002
13	9/7/13/3/6/6/10/7	3 (0)	2	1997, 2002
14	NA	2 (0)	2	2000
15	NA	2 (0)	2	1998, 2000
16	NA	2 (0)	2	1991
17	NA	2 (0)	2	1995, 1997
18	NA	2 (0)	2	1998, 2000
19	NA	2 (0)	2	2001, 2003
Singletons	–	66 (0)	60	All years except 1990, 1995, and 2004

*MLVA, multilocus variable number tandem repeat analysis; NA, not applicable because cluster comprises <3 strains. Singletons, MLVA profiles that are not included in a certain cluster. MLVA profiles are defined by repeat numbers after the following variable number tandem repeat (VNTR) locus order: 3/34/9/25/17/19/36/37 with EDL933 as standard with the following MLVA profile: 9/10/11/5/6/6/8/7 (based on genome sequence analysis). Strains belonged to 1 cluster if they differed at <2 VNTR loci.

**Figure:**
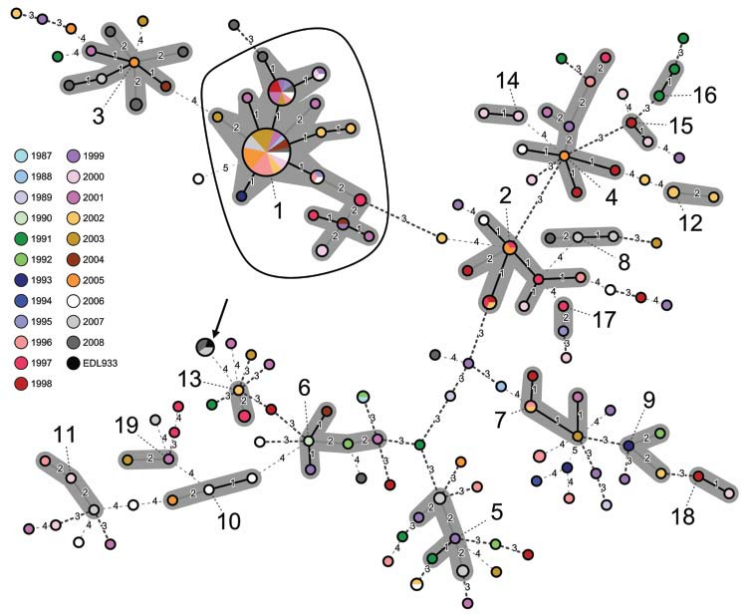
Minimum spanning tree based on multilocus variable number tandem repeat analysis (MLVA) allelic profiles, showing the phylogenetic relationship between 202 epidemiologically unrelated enterohemorrhagic *Escherichia coli* (EHEC) O157:H7/H^–^ strains, Germany, 1987–2008. Each node represents a unique MLVA profile. Size of the nodes is proportional to the number of isolates per MLVA profile. Small numbers and specific dashed lines between nodes represent distance between the nodes, i.e., number of different alleles. Clusters are indicated by numbers according to their size (Table 2) and are highlighted in gray. All strains within the enclosed area are sorbitol-fermenting EHEC O157:H^–^. The arrangement of colors within each node reflects the proportion of strains isolated from different years per node and corresponding MLVA profile. Arrow indicates reference strain *E. coli* O157:H7 EDL933.

All 61 strains in cluster 1 were SF EHEC O157:H^–^. The remaining 141 strains did not ferment sorbitol (Table 2, Figure). The phylogenetic relationship of 202 EHEC O157 strains based on the 141 MLVA profiles is shown in an MST in the Figure. The reference strain EDL933, which is also included in the MST, shares its MLVA profile with 4 isolates from Germany obtained during 2007–2008.

### Association of MLVA Profiles with HUS

To determine whether there was an association between MLVA profiles or clusters and the ability of these strains to cause HUS, we performed a significance test. The 2 most common MLVA profiles were significantly associated with HUS (p = 0.023). Testing for specific clusters resulted in a significant association of HUS with cluster 1 (p = 0.009) (Figure).

## Discussion

Using 8 VNTR loci of the current PulseNet MLVA O157 protocol (*23*), we analyzed a large collection of 202 EHEC O157:H7/H^–^ strains isolated over >2 decades in Germany to determine their molecular epidemiology. Of the 141 MLVA profiles detected, 81 were clustered into 19 groups of related profiles that differed at >2 loci. The remaining 60 profiles were not clustered. Our data demonstrate a great diversity of EHEC O157:H7 associated with human diseases in Germany over the past 2 decades. The wide distribution of strains within the MST based on MLVA typing reflects frequent occurrence of genetic events outside the EHEC O157 core genome (Table 2, Figure). The 19 MLVA clusters included 67.3% (136/202) of the analyzed strains. Further analysis of the clusters including >4 MLVA profiles did not show any specific clustering of strains in time. Most of the larger clusters (clusters 1, 2, 4, 5, and 6; Table 2) contained strains widespread in the period of 10–20 years. Only cluster 3 is defined by profiles starting from 2001, which indicates a later appearance than clusters 1, 2, 4, 5, and 6 (Table 2).

The 2 most frequently identified MLVA profiles are parts of cluster 1, which indicates a consensus profile among SF EHEC O157:H^–^ isolates over time within this cluster. The corresponding strains include strain 493/89, which was isolated during the first documented outbreak caused by SF EHEC O157:H^–^ (*25*). All other isolates that exhibited the 2 most common MLVA profiles also fermented sorbitol, which identified strain 493/89 as a prototype of these strains. This finding corroborates the assumption of an epidemic bacterial population structure within a background population comprising a network between different genotypes, and that superimposed strains emerge from highly adaptive, ancestral genotypes and may be persistent for decades (*35*). Nodes from cluster 1, which represent the 2 most prevalent MLVA profiles, include strains from 1988–2008 and 1995–2008. This finding indicates a persistence of these successful clones, which supports this hypothesis. Moreover, evolutionary success and uniqueness of this SF clone was recently supported by whole genome single nucleotide polymorphism analysis, in which distinct branching of these clones was determined during evolution of the O157 serotype (*22*).

Statistical analysis demonstrated that the 2 most common profiles and the entire cluster 1 are associated with HUS, which indicates that specific MLVA profiles are associated with severe disease. Cluster 1 comprised 61 of the strains and was distributed over more than a decade. Although not statistically significant, 10 of 11 isolates in cluster 2 were also associated with HUS. Despite these similarities, they exhibited different MLVA profiles (Table 2; Figure). Extensive heterogeneity of EHEC O157:H7, in contrast to conservation of SF EHEC O157:H^–^, could be related to observed differences in the nature of the reservoirs and vehicles for transmission. In addition, the epidemiology of SF EHEC O157:H^–^ infections differs markedly because these infections occur predominantly during cold (winter) months and in children <3 years of age (*5*). Moreover, although EHEC O157:H7 infections have zoonotic origins, SF EHEC O157:H^–^ are rarely found in animals (*36*). Humans are plausibly the main reservoirs, as is the case with classical enteropathogenic *E*. *coli* and enteroinvasive *E*. *coli*. This relatively stable niche may lead to the conserved genome structure and high pathogenicity for the host (*37*).

Four strains isolated in 2007 and 2008 exhibited the same MLVA profile as the reference strain EDL933 isolated in 1982 in the United States (*38*) (Table 2, Figure). Among the 3,200 entries in the CDC MLVA database, the EDL933 MLVA profile was detected only during an outbreak in 1982 (E. Hyytiä-Trees, pers. comm.). There are 2 possible explanations for this phenomenon. This finding is coincidental because of genetic changes in the O157 genome or EDL933 shares a common MLVA profile with other strains. The presence of such common profiles is known, especially in foodborne pathogens and other monomorphic species (*39*) and frequently seen by using other typing techniques, such as PFGE.

Analysis of the number of alleles of different VNTRs produced results similar to those of a previous study (*8*). Whereas the ID was high (0.74–0.90; Table 1) in VNTR loci consisting of 6–7-bp repeats (all VNTR loci except VNTR-34), the ID was low (0.66) for the 18-bp repeat (VNTR-34). Whether a VNTR locus is located within an open reading frame did not influence the ID (Table 1). However, the frequency of null alleles differed markedly. A total of 98 (48.5%) of 202 strains exhibited null alleles in 4 of the 8 VNTR loci. Especially in VNTR-9 and VNTR-36, the frequency of null alleles was high (31.7% and 44.1%). Although null alleles were reported in other MLVA O157 studies (*8*,*40*), this high frequency of null alleles determined in our study might indicate a specific feature of EHEC O157 strains from central Europe or Germany. An explanation for the frequent occurrence might be that VNTR-36 and VNTR-9 are located in noncoding or hypothetical protein encoding regions of the EHEC genome (Table 1). Nevertheless, all strains had a high ID regarding the complete MLVA profile.

Our study had some limitations. Because of the limited number of isolates obtained during 1987–1995, clustering might be biased and a more year-specific clustering might be observable. However, cluster 1 represents 30.2% (61/202) of strains widespread during 1988–2008, which contradicts this thesis, and infers a certain genetic stability of such clusters over time. In contrast to phylogenetic studies based on whole genome sequencing data (*22*), we report a phylogeny based on 8 genetic loci that might be biased by larger recombinational events. However, all VNTR loci are >50 kb from the *rfb-gnd* segment, which was determined to be the only genomic region in EHEC O157 with a higher mutation rate (*22*).

Strains (66/202, 32.7%) that were not classifiable into any MLVA cluster complement the assumption of the highly dynamic EHEC O157 genome. This finding likely indicates that genetic changes in *E*. *coli* lead to adaptation to a host-specific environment (in this case human), especially during pathogenesis and host-specific immune responses.

Applying MLVA to this highly diverse strain collection resulted in new insights into the phylogeny of EHEC O157 in Germany since their first description in 1987. In addition to its already demonstrated ability to differentiate outbreak and sporadic case strains, MLVA of O157 emerged as a major typing tool that can further characterize EHEC O157 subpopulations and associated strains. This tool can be used for studying phylogeny coherences and identifying successful clones.

## Appendix

**Table A1:** PCR primers and fluorescent dyes used for analysis of VNTR loci of enterohemorrhagic *Escherichia coli* O157, Germany, 1987–2008*

Locus†	Forward primer (5′ → 3′) and labeled dyes‡	Reverse primer (5′ → 3′)	Final concentration, μmol/L§
Multiplex 1			
VNTR-3	NED-GGCGGTAAGGACAACGGGGTGTTTGAATTG	GAACAACCTAAAACCCGCCTCGCCATCG	0.025
VNTR-9	6-FAM-GCGCTGGTTTAGCCATCGCCTTCTTCC	GTGTCAGGTGAGCTACAGCCCGCTTACGCTC	0.05
VNTR-25	VIC-GCCGGAGGAGGGTGATGAGCGGTTATATTTAGTG	GCGCTGAAAAGACATTCTCTGTTTGGTTTACACGAC	0.05
VNTR-34	6-FAM-GACAAGGT TCTGGCGTGTTACCAACGG	GTTACAACTCACCTGCGAATTTTTTAAGTCCC	0.05
Multiplex 2			
VNTR-17	NED-GCAGTTGCTCGGTTTTAACATTGCAGTGATGA	GGAAATGGTTTACATGAGTTTGACGATGGCGATC	0.05
VNTR-19	6-FAM-GCAGTGATCATTATTAGCACCGCTTTCTGGATGTTC	GGGGCAGGGAATAAGGCCACCTGTTAAGC	0.05
VNTR-36	6-FAM-GGCGTCCTTCATCGGCCTGTCCGTTAAAC	GCCGCTGAAAGCCCACACCATGC	0.025
VNTR-37	VIC-GCCGCCCCTTACATTACGCGGACATTC	GCAGGAGAACAACAAAACAGACAGTAATCAGAGCAGC	0.0125

*VNTR, variable number tandem repeat. Adapted from Hyytiä-Trees et al. (*23*). †Primers are listed as PCR mixtures. ‡The 5′ end of the forward primer is labeled in each case. §Both multiplex PCRs were performed in a volume of 10 μL. Thermal cycling reactions consisted of an initial denaturation (5 min at 95°C); 28 cycles of denaturation (30 s at 95°C), annealing (90 s at 60°C), and extension (30 s at 72°C); and a final extension (30 min at 60°C).
